# Qualitative and Quantitative Characteristics of Organic Acids in Monofloral and Honeydew Honeys from Poland: Is There a Varietal Pattern in Their Composition?

**DOI:** 10.3390/molecules30214261

**Published:** 2025-10-31

**Authors:** Teresa Szczęsna, Katarzyna Jaśkiewicz, Jacek Jachuła

**Affiliations:** Apiculture Division, The National Institute of Horticultural Research, Konstytucji 3 Maja 1/3, 96-100 Skierniewice, Poland; katarzyna.jaskiewicz@inhort.pl (K.J.); jacek.jachula@inhort.pl (J.J.)

**Keywords:** honey, botanical origin, organic acids, HPLC-DAD, lactic acid, PCA

## Abstract

Organic acids shape the organoleptic properties of honey and are linked to its health-beneficial properties. Their composition is affected by botanical and geographical origin, and some acids have even been proposed as markers for authentication of varietal honeys. Organic-acid composition was determined using high-performance liquid chromatography with photodiode-array detection (HPLC-DAD) in 152 samples of monofloral (willow, acacia, rape, phacelia, linden, heather, buckwheat and goldenrod) and honeydew (deciduous and coniferous) honeys from Poland. The deciduous and coniferous honeydew honeys were distinguished by high content of L-(+)-lactic acid and the presence of succinic acid as well as high total content of acids. Buckwheat honey was the only variety for which the presence of D-(−)-tartaric acid was quantified. These three honey varieties were clearly separated from the others using principal component analysis (PCA). Samples from the other varieties formed one cluster. We conclude that while some promising results were obtained for distinguishing honeydew and buckwheat honeys from other varieties, further investigation is needed, including analysis of additional acids and possibly other physicochemical parameters.

## 1. Introduction

Organic acids in honey are mainly derived from nectar and/or honeydew but can also be products of glucose and fructose metabolism during maturation of honey [1,2]. These are principally the following acids: gluconic, citric, pyruvic, malic, fumaric, maleic, tartaric, lactic, propionic, quinic, hydroxypropionic, propanodic, adipic, suberic, succinic, butyric, acetic, lactic, galacturonic, tartronic, α-ketoglutaric, trans-aconitic and azelaic [3,4,5,6,7,8,9,10,11,12,13].

The analysis of organic acids in honey is of great interest to the food industry. These components, together with other constituents—primarily sugars—play a key role in determining the sensory properties, purity and authenticity of honey [14]. They are also responsible for the antibacterial and antioxidant properties of honey [15]. Additionally, organic acids serve as indicators of honey quality [4,16]. During prolonged storage or under improper conditions, fermentation processes may occur, resulting in the formation of volatile organic acids, especially acetic acid, which increases markedly because yeasts metabolize honey sugars into ethanol that is further oxidized to acetic acid. Therefore, elevated acetic acid concentrations can signal initiated or ongoing fermentation, being a chemical marker of honey spoilage and quality loss [17].

Substantial differences in the concentrations of these acids among honey varieties have been reported. Therefore, the analysis of the qualitative and quantitative composition of organic acids in honey is regarded as useful to determine the botanical and geographical origins of honeys. Wilkins et al. [18] identified 32 different organic dicarboxilic acids in honeys originating from New Zealand. Succinic and glutaric acids, as well as their methyl esters, were found to be characteristic for *Knightea excelsa* honey. A study by Haroun et al. [5] on organic acids in nectar and honeydew honey originating from Turkey showed that honey from pine (*Pinus* spp.) and chestnut (*Castanea sativa*) honeydew were characterized by a high content of tartaric, malic, maleic, citric, succinic and fumaric acids compared with other tested honeydew and nectar honeys. Cotton (*Gossypium barbadense*) and mountain vegetation honey contained only fumaric, malic and citric acids. Those authors suggested malic acid as a marker for cotton honey. That same study also confirmed reports from other authors [19,20,21] that oak honeydew contains more citric acid (87.22–142.28 mg/100 g) than nectar honeys (55.64–85.54 mg/100 g). Sun et al. [12] identified and determined 22 organic acids in five varieties of honey (acacia, jujube, vitex, canola, rapeseed and linden) and found significant differences between the studied honey varieties. They found the highest content of organic acids in jujube honey (85.07 mg/100 g), while the lowest was in acacia honey (40.91 mg/100 g). Gluconic acid dominated in bracatinga honey (*Mimosa scabrella*), accounting for 55 to 69% of the total organic-acid content of that variety of honey and in manuka honey, constituting 65 to 100% of the total organic-acid content [11,13,22,23].

Although different honey varieties may exhibit varying levels of a given metabolite, this alone does not establish it as a reliable marker for a particular variety. Recent studies therefore have considered multiple physicochemical traits of honey and used multivariate analyses, e.g., principal component analysis (PCA), or further developed machine learning algorithms for the authentication of the botanical origins of honeys [24]. For example, honeydew honey from bracatinga (*Mimosa scabrella*) contains a higher content of citric acid compared with nectar honeys; however, to distinguish these two types of honey, in addition to citric acid, other organic acids, such as gluconic, succinic, glycolic, glutaric, malic, acetic and lactic acid, should be taken into account [11,22].

It cannot be ignored that the presence of organic acids in honey can also be due to honey bee treatment against *Varroa* mites. Bogdanov et al. [25] showed that the content of formic acid in honey, after its application in bee colonies increases and accumulates to a higher extent in the honey after the spring treatment, affects honey taste. By contrast, there was no increase in oxalic acid concentration after its use in honey bee colonies. No significant changes in oxalic acid content in honey after treatment were reported by Thurston et al. as well [26].

High-performance liquid chromatography coupled with a diode-array detector (HPLC-DAD) is the most widely employed technique for the detection and quantification of organic acids in honey [3,5,6,7,8,10,20,27]. This method enables the separation of individual acids and their simultaneous measurement across multiple wavelengths, providing both qualitative (spectral) and quantitative (concentration) information. HPLC-DAD is recognized for its high precision, sensitivity and capacity to quantify multiple organic acids within a single analysis. To achieve optimal resolution of organic acids in honey, various experimental parameters have been optimized, including the use of ion-exclusion columns specifically designed for organic-acid separation in different matrices, as well as adjustments to mobile-phase composition, pH and temperature [20,27].

So far, studies on varietal honeys produced under Polish climatic conditions have not addressed the content of naturally occurring organic acids. Therefore, the present study focuses on the qualitative and quantitative composition of organic acids in monofloral and honeydew honeys from Poland. A total of 152 Polish honey samples were analyzed, representing the willow, acacia, rapeseed, phacelia, lime, heather, buckwheat, goldenrod, leafy honeydew and conifer honeydew varieties. Prior to the analyses, the HPLC-DAD method was developed and validated to ensure the reliability of the results. The relationship between botanical origin and acid composition was examined using multivariate statistical analyses. Understanding the organic acid profiles of varietal honeys may provide a valuable foundation for further research on how these compounds influence the physicochemical and sensory properties of honey, as well as stability during storage.

## 2. Results and Discussion

### 2.1. Botanical Origins of Honey Samples

Melissopalynological analysis of the honey samples tested allowed them to be classified into the following varieties: willow, acacia, rape, phacelia, lime, heather, buckwheat and goldenrod (Table 1). Specific pollen content showed variability from 9.1% (for rape honey) to 23.9% (for linden honey). The minimum percentage of predominant pollen required for the classification of monofloral honeys by legislation varies between countries [28]. The most prominent example is the proportion of *Fagopyrum* pollen, for which certain international standards specify a minimum threshold of 30%, whereas Polish regulations stipulate a minimum of 45% for classification as buckwheat honey. Requirement differences may result in inconsistent classification of varietal honeys and consequently affect the organic-acid composition of the varietal honeys tested. The shares of pollen of other plant species in honeys classified as varietal also influences the chemical profiles [29,30].

Honeydew honey presents higher electrical conductivity than nectar honeys, being a good parameter to differentiate both types of honey. The honey samples were classified as deciduous honeydew honey if the electrical conductivity was between 0.80 and 0.95 mS/cm and as coniferous honeydew honey if the electrical conductivity was over 0.95 mS/cm. The mean value of the electrical conductivity for the deciduous honeydew honey samples was 0.91 ± 0.07 mS/cm, and for coniferous honeydew honey samples, it was 1.12 ± 0.14 mS/cm. The results of the electrical conductivity of the Polish honeydew honeys obtained in the study presented in this paper are comparable with those obtained in our other study [31].

**Table 1:** Results of melissopalynological analysis and electrical conductivity measurements of the studied honeys.

	**Specific Pollen Content**				
**Honey Variety**	**Min–Max (%)**	**Mean (%)**	**Standard** **Deviation**	**Coefficient of** **Variation (%)**	**Requirements of the Minimum Percentage of Pollen/Electrical Conductivity** **(According to PN-88/A-77626 “Miód pszczeli”, 1988 [32])**
Goldenrod— *Solidago* spp. (*n* = 5)	48.0–81.0	67.1	10.3	15.4	45 *
Willow— *Salix* spp. (*n* = 9)	48.0–67.0	56.3	8.5	15.1	45 **
Acacia— *Robinia pseudoacacia* (*n* = 9)	30.9–40.7	33.2	4.8	13.4	30
Buckwheat— *Fagopyrum esculentum* (*n* = 14)	49.9–90.5	61.2	12.7	20.7	45
Linden—*Tilia* spp. (*n* = 23)	20.1–49.0	39.3	9.4	23.9	20
Phacelia— *Phacelia tanacetifolia* (*n* = 32)	47.0–90.0	72.7	13.9	19.2	45 ***
Rape— *Brassica napus* (*n* = 29)	69.0–96.0	87.1	7.9	9.1	45
Heather— *Calluna vulgaris* (*n* = 5)	49.0–72.0	64.1	8.0	12.4	45
**Electrical Conductivity (mS/cm)**					
Coniferous honeydew (*n* = 15)	0.95–1.45	1.12	0.14	12.6	0.95
Deciduous honeydew (*n* = 11)	0.80–0.93	0.91	0.07	7.6	0.80

* According to [33,34,35]. ** According to [36,37,38]. *** According to [39,40,41].

### 2.2. HPLC-DAD Method Validation

Effective chromatographic separation and quantitation of 12 commonly found organic acids (oxalic, D-(−)-tartaric, D-(−)-quinic, formic, D-(+)-malic, malonic, L-(+)-lactic, citric, fumaric, succinic, maleic and propionic acid) in honey was achieved using the Shimadzu HPLC system with a DAD detector (Figure S1). Chromatographic separation was achieved in under 15 min. Table 2 shows the validation parameters of the HPLC-DAD method for organic acid determination in honey. The results exhibited good repeatability and linearity over the tested concentration ranges. The LOD and LOQ values of the 12 organic acids ranged between 0.01 and 4.45 µg/mL and between 0.03 and 13.50 µg/mL, respectively. Compared with the others, the LOD of the maleic acid was the lowest, whereas that of the propionic acid was the highest. The coefficients of determination (R^2^) for all analyzed organic acids ranged from 0.9994 to 1.0000 and the recovery rates from 91.42% (D-(+)-malic acid) to 98.68% (succinic acid).

To maximize the resolution of organic acids in honey, various experimental parameters were tested in other studies, including the use of ion-exclusion columns designed for organic-acid separation in different matrices as well as adjustments to mobile phase composition, pH and temperature [3,5,6,7,8,10,20,27]. Several mobile phases were evaluated, such as sulfuric and metaphosphoric acid at different concentrations and pH levels under isocratic conditions. Additionally, a mixture of acetonitrile and potassium phosphate under gradient conditions was also employed [6]. However, it is not possible to directly compare the validation methodology of the present HPLC method with the literature data, as this is the first study to apply a Synergi Hydro-RP 80Å C18 column (250 × 4.6 mm, 4 µm; Phenomenex Inc., Torrance, CA, USA) for the analysis of organic acids in honey using 20 mM potassium phosphate (pH 2.9) as the mobile phase. Extremely polar analytes are often poorly retained and insufficiently separated on conventional C18 columns. The Synergi Hydro-RP column, however, features a C18 bonded phase endcapped with a proprietary polar group, providing enhanced retention and separation of both hydrophobic and polar compounds under 100% aqueous conditions—making it particularly well-suited for the analysis of organic acids.

### 2.3. Organic-Acid Composition of Monofloral and Honeydew Honeys

So far, to the best of our knowledge, no studies have investigated the qualitative and quantitative compositions of non-phenolic organic acids in monofloral and honeydew honeys from Poland. Therefore, we focused on the 12 acids that were most frequently and abundantly identified in honey samples of various botanical and geographical origins, as reported by previous authors from other regions of the world [3,5,6,7,8,10,20,27]. We are, however, well aware that numerous other acids have also been detected in honey: e.g., glycolic, glucuronic, 3-hydroxypropionic, propanediolic, benzoic, adipic, α-ketoglutaric, tartronic, suberic, shikimic, butyric, isobutyric, pyruvic, citramalic, ascorbic and gluconic acids [12,42]. It should also be noted that the analytical capabilities of our laboratory posed a certain limitation in the study of organic acids in honey. The available instrumentation allowed for the determination of only the 12 selected acids, for which the validation parameters—limit of detection and quantification, linearity range, precision (CV) and accuracy (expressed as recovery in the HPLC assay)—were found to be satisfactory.

The contents of the 12 organic acids in the Polish monofloral and honeydew honeys are presented in Table 3. Oxalic, D-(−)-quinic, formic, citric and propionic acids were quantified in all honey varieties.

The oxalic acid content ranged from 15.31 mg/kg in rape honey to 29.31 mg/kg in deciduous honeydew honey. Our results for the acacia (17.13 mg/kg) and linden (19.60 mg/kg) honeys are comparable with those reported by Ciucure & Geană [3] and Sun et al. [12] for these two monofloral honeys, whereas the values obtained for honeydew honey (both deciduous and coniferous) were much lower compared with the reported mean value of 106.16 mg/kg. The oxalic acid content determined in Spanish oak honeydew honey was higher, 63.9 mg/kg on average [27]. Higher values were also obtained by those authors for heather (*Erica* sp.) and ling (*Calluna vulgaris*) honeys: over 100 mg/kg on average. Mato et al. [23] also marked high oxalic acid content in chestnut and multifloral honeys, with a mean value of 40.4 mg/kg. Other authors did not detect oxalic acid in honeys from different botanical origins and regions of Turkey, Saudi Arabia or Latvia [6,8,43]. Some authors suggest that oxalic acid, when in high concentrations, indicates acid after anti-*Varroa* bee treatment [3,12,27], though Bogdanov et al. [25] and Thurston et al. [26] did not confirm the increase in oxalic acid content in honey after the treatment. Moosbeckhofer et al. [44] confirmed the presence of oxalic acid in honey from non-treated colonies, which suggests the natural presence of the acid in honey.

D-(−)-quinic acid was quantified in the highest amounts in deciduous and coniferous honeydew honey (182.73 and 140.39 mg/kg, respectively), while the lowest content (41.13 mg/kg) was found for goldenrod honey. Compared with our results, much lower concentrations of quinic acid in monofloral honey (lime, vitex, canola, acacia, jujube) were detected by other authors (from 0.38 mg/kg in canola honey to 12.67 mg/kg in linden honey) [12]. Hrobonová et al. [45] did not detect quinic acid in Slovak honey. However, the detection limit (10 µg/mL) and quantification limit (30 µg/mL) reported in their study were considerably higher than those achieved in ours. A large range of concentrations of this acid (20–4470 mg/kg) was determined by Keke and Cinkmanis [6] to be in multifloral honeys purchased from the Latvian market.

Formic acid concentrations ranged from 30.48 mg/kg in acacia honey to 100.76 mg/kg in deciduous honeydew honey. High concentrations (>75 mg/kg) of this acid were also found in coniferous honeydew, buckwheat and heather honey. The results for formic acid content in different honey varieties vary widely [7,23,43,46,47,48]. Formic acid was the predominant organic-acid (5.7–15.7 mg/kg) in the three honey types (citrus, clover and cotton) from Egypt [7]. In Saudi Arabian honeys from different botanical sources and regions, formic and malonic acids were found in the highest concentrations [8]. The formic acid content in Spanish honeys varied over the range of 27.5 (rosemary honey) to 150.6 mg/kg (heather honey) [44]. In honeydew honey, the formic acid content amounted from 80 to 110 mg/kg [43,49]. Despite being regarded as a natural compound of honey, the presence of formic acid in honey may be a result of anti-*Varroa* treatment. It was shown that the content of formic acid in honey, after its application in bee colonies, increased but was within the range established for honey from apiaries where this compound was not used [25,50].

The highest citric acid concentrations were detected in the honeydew honeys (22.52–44.90 mg/kg), buckwheat honey (37.61 mg/kg) and rape honey (25.55 mg/kg), whereas the lowest levels occurred in the phacelia (8.86 mg/kg) and acacia (3.77 mg/kg) honeys. Overall, the results for Polish monofloral honeys fall within the range reported for honeys of different geographical origins, including China, Japan, Spain, Romania, Canada, the USA, Argentina and New Zealand [13]. The citric acid content in the deciduous honeydew honey in our study was comparable with that reported for bracatinga honeydew honey from Brazil [11]. Similarly, the values obtained for coniferous honeydew honey were close to those found in *Quercus* spp. honey from Spain, though considerably lower than the results for Turkish honeydew honeys [5,27]. By contrast, much higher citric acid concentrations (250–280 mg/kg) in honeydew honey were reported by Ohmenhaeuser et al. [49]. Much higher citric acid levels have also been documented in Turkish chestnut honey (78.9–465.0 mg/kg) and pine honey (124.0 mg/kg) [43], as well as in Galician (northwestern Spain) chestnut honey (158–394 mg/kg), while eucalyptus and citrus honeys were characterized by low citric acid content [20,23]. The concentration of the acid in Saudi Arabian honeys was at a very low level (below 0.5 mg/kg) [8]. In a study by Tezcan et al. [43], in rhododendron honey, citric acid was detected but not quantified, and in acacia honey, this acid was not detected at all. Citric acid was also not detected in Egyptian clover or cotton honey [7]. In contrast to the very low citric acid content found in acacia honey in our study, Sun et al. [12] reported considerably higher levels, reaching 45 mg/kg. Differing from our results, Ohmenhaeuser et al. [49] did not detect citric acid in rape honey.

In our study, the propionic acid concentration ranged from 3.01 mg/kg in rape honey to 82.49 mg/kg in buckwheat honey. In addition to buckwheat honey, acacia honey was characterized by high content of this acid (48.24 mg/kg). Relatively high amounts of propionic acid were identified in acacia, rape, sunflower, lime and honeydew honey and ranged from 67.92 (for lime honey) to 93.98 mg/kg (for sunflower honey), as reported by Ciucure and Geana [3]. Much lower content of this acid in acacia honey (32.0 mg/kg) was quantified by Paulic et al. [10]. Other authors also found very low values for some varieties of monofloral honey—citrus and clover Egyptian honeys (0.63 and 0.40 mg/kg, respectively) [7] Similar low levels of propionic acid were reported in monofloral honeys from Saudi Arabia, ranging from 0.01 to 0.7 mg/kg [8]. In a study by Bergamo et al. [51], propionic acid was not detected in multifloral honeys. Concentrations of this acid in honeydew honeys from Spain (*Quercus robur*, *Quercus ilex*) ranged from 33.7 to 65.7 mg/kg [27]. The results received in our study for honeydew honey were more than twofold lower.

D-(+)-malic acid was predominant in the buckwheat, deciduous and coniferous honeydew honeys (mean in the range of 86.21–233.28 mg/kg). The concentrations of this acid in other honeys were below 30 mg/kg. The D-(+)-malic acid in heather honey was below the limit of detection. The results presented in our paper for malic acid content in linden and acacia honey are much lower than the results received for the same varieties by Sun et al. [12]: 61.06 and 19.09 mg/kg, respectively. The malic acid content obtained in our study for buckwheat honey (86.21 mg/kg) was comparable with the value determined by the same authors for jujube honey (72.46 mg/kg). Very high content of malic acid was detected by other authors in oak honeydew honey (1134.3 mg/kg) and cotton and chestnut honey (ranging from 415.1 to 1806.4 and from 170.3 to 655.4 mg/kg, respectively) [5].

Coniferous honeydew contained the highest amount (2162.49 mg/kg) of L-(+)-lactic acid, followed by deciduous honeydew honey (854.18 mg/kg). The level of L-(+)-lactic acid in honeydew honeys determined in our study is much higher compared with the literature data [4,11,12,22,23,46]. Higher concentrations of lactic acid have been found in bracatinga honeydew honey compared with blossom honey [11,22], and lactic acid, together with glycolic, glutaric, malic, acetic and gluconic acid contents, was used to build a classification model to distinguish bracatinga honeydew honey from blossom honeys and adulterated bracatinga honeys. The L-(+)-lactic acid content determined in acacia honey (36.91 mg/kg) in our study was comparable with the value obtained by Sun et al. [12]. The content of this acid in rape honey (18.93 mg/kg) in our study was much lower compared with the results of those authors (82.36 mg/kg). Those authors also detected relatively high levels (64.73 mg/kg) of L-(+)-lactic acid in linden honey, while in our study, the amounts of this acid in linden honey were below the limit of detection. Lactic acid is present in honey mainly because of the activity of lactic acid bacteria (LAB) present in floral nectar and the honey bee digestive tract. The high content of lactic acid in honeydew honey can be explained by the presence of the acid already in the honeydew excreted by sap-feeding insects, likely because of the activity of LAB colonizing the digestive tracts of these insects [4,11,52,53,54].

We detected and quantified fumaric acid in all honey varieties, except in acacia honey, and the acid concentration was within a similar range to those reported by other authors for monofloral, honeydew and multifloral honeys [3,12,20]. Higher content of the acid (ca. 10 mg/kg) was found in some of the multifloral honeys from the Latvian market [6], as well as in the Turkish floral honeys (sunflower, multifloral, cotton, chestnut) and in the honeydew honeys (pine, oak) [5]. Pine honey was particularly rich in fumaric acid, with values ranging from 6.4 to 56.7 mg/kg.

Maleic acid was detected in all honey varieties except acacia, at very low concentrations ranging from 0.01 to 0.10 mg/kg. Only a few publications in the literature report on the presence of maleic acid in honey. The data presented in these papers also show that the concentration of maleic acid was on a very low level. Our results are consistent with those obtained by Nafea et al. [8] and Ciucure & Geana [3]. However, Mohandes and Sawsan [7] reported higher quantities of the acid: 1.11 mg/kg in clover honey and 5.68 mg/kg in cotton honey. Furthermore, Brugnerotto et al. [22] quantified much higher concentrations of maleic acid in bracatinga honeydew honey (409–791 mg/kg) and nectar honeys (90.0–143 mg/kg) using capillary electrophoresis.

Malonic acid was detected only in linden, heather and rape honey. Its concentration was much higher in linden (63.65 mg/kg) and heather honey (60.38 mg/kg) compared with rape honey (1.85 mg/kg). The available literature provides only limited data on malonic acid in honey. This acid was quantified in citrus, clover and cotton honey in the range of 0.70 to 8.00 mg/kg [7]. The concentration of malonic acid in the Saudi Arabian honey types was even lower (0.1–0.3 mg/kg) [8]. In contrast, Brugnerotto et al. [22], using capillary electrophoresis, found much higher contents of malonic acid in nectar (822–1340 mg/kg) and bracatinga honeydew (2590–3300 mg/kg) honeys from Brazil.

Succinic acid was present only in the honeydew honeys, with a concentration of 10.82 mg/kg in coniferous honeydew honey and of 19.47 mg/kg in deciduous honeydew honey. Succinic acid was determined to be in nectar honey varieties and in honeydew honey by many authors. Generally, honeydew honey and chestnut honey are characterized by high amounts of this acid [5,11,19,20,43,49]. The results reported by those authors substantially exceed the levels observed in our study. According to them, the concentration of succinic acid varied from 170.0 to 1329.0 mg/kg in honeydew honey and from 39.0 to 648.8 mg/kg in chestnut honey. Brugnerotto et al. [22] reported even higher concentrations of succinic acid (4840 mg/kg to 6720 mg/kg) in bracatinga honeydew honey. In contrast to our study, the studies of del Campo et al. [46] and Sun et al. [12] reported the presence of succinic acid in many varieties of nectar honey, such as linden, rape, acacia, vitex, jujube, eucalyptus, heather, lavender, orange, thyme and rosemary, in the range of 7.1 (orange blossom) to 69.7 mg/kg (eucalyptus). Suto et al. [13] reported the succinic acid contents in 25 commercial and Manuka honeys from several countries (China, Japan, Spain, Romania, Canada, USA, Argentina and New Zealand) to be from 4.9 to 91.9 mg/kg.

D-(−)-tartaric acid was detected exclusively in buckwheat honey, with a mean concentration of 22.17 mg/kg. Our results were much higher compared with the results obtained by other authors. Brugnerotto et al. [22] did not detect tartaric acid in any of their bracatinga honeydew or nectar honey samples. In the study by Sun et al. [12], the concentration values of tartaric acid in linden, vitex, canola, acacia and jujube honeys were very low and ranged from 0.24 mg/kg (canola honey) to 1.14 mg/kg (acacia, jujube honey). Suto et al. [13] reported the presence of tartaric acid in only 5 of the 25 commercially available honeys from various countries (China, Japan, Spain, Romania, Canada, USA, Argentina and New Zealand), with concentrations ranging from 2.8 to 15.0 mg/kg.

Coniferous honeydew honey and deciduous honeydew honey had the highest total content of organic acids (2597.48 mg/kg and 1492.09 mg/kg, respectively). In the group of monofloral honeys, buckwheat honey had the highest total organic-acid content (499.00 mg/kg, on average), while the values in the other varieties were in the range of 156.84–270.78 mg/kg. Content of organic acids strongly contributes to honey taste, acidity, pH and electrical conductivity [22]. Honeydew honeys are generally characterized by higher content of organic acids and higher free acidity than nectar honeys [11,55,56,57].

### 2.4. Hierarchical Clustering and PCA

**Figure 1:**
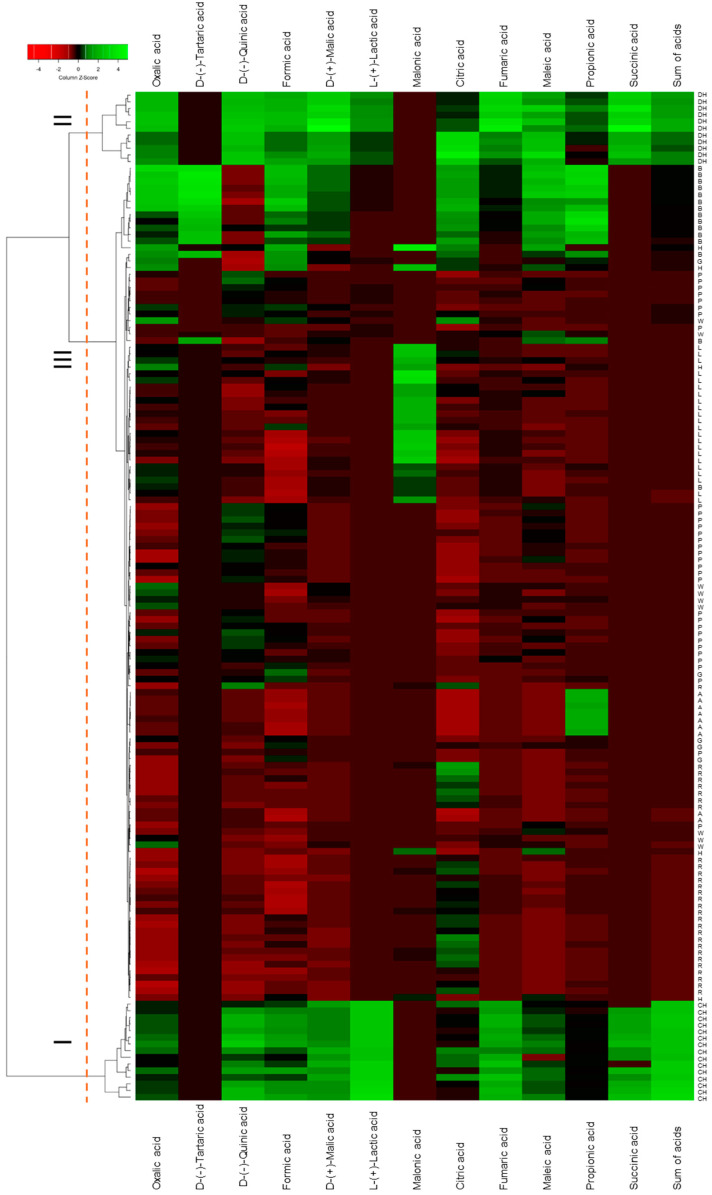
Dendrogram of hierarchical cluster analysis made combined with heatmaps for monofloral and honeydew honey samples. G—goldenrod; W—willow; A—acacia; B—buckwheat; L—linden; P—phacelia; R—rape; H—heather; CH—coniferous honeydew; DH—deciduous honeydew.

Hierarchical clustering analysis was visualized as a dendrogram combined with a heatmap (Figure 1). Coniferous honeydew honeys form cluster I, characterized by the highest concentrations of D-(−)-quinic, formic, D-(+)-malic, fumaric and L-(+)-lactic acids and total organic acids; this variety was also one of two in which succinic acid was detected. Deciduous honeydew honeys form cluster II, distinguished by elevated concentrations of D-(−)-quinic, formic, D-(+)-malic, citric, fumaric, L-(+)-lactic and maleic acids, as well as the presence of succinic acid. Cluster III comprises all other samples, further divided into subclusters according to malonic, D-(−)-tartaric or propionic acid content. Twelve of the fourteen buckwheat honeys were grouped into a single subcluster; the linden honeys formed another separate one, defined by the highest malonic acid content; and acacia honeys were distinguished by the highest propionic acid levels.

**Figure 2:**
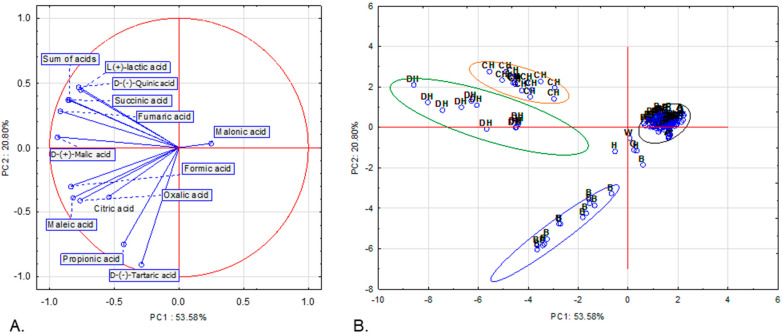
Projection of variables (**A**) and cases (**B**) in two-factor plane (PC1 × PC2). G—goldenrod, W—willow, A—acacia, B—buckwheat, L—linden, P—phacelia, R—rape, CH—coniferous honeydew, DH—deciduous honeydew, H—heather honey.

We further employed principal component analysis (PCA) using the same 13 variables (contents of each of the 12 organic acids and the total content of acids) as in the hierarchical clustering to identify similarities between the samples in terms of organic-acid composition. Three principal components, with eigenvalues ≥ 1, explained 82.37% of the total variance (Table S1). Figure 2A shows the projection of the variables on a two-factor plane, PC1 × PC2. The first principal component (PC1), accounting for 53.58% of the variance, shows negative correlations with all variables, with the exception of malonic acid content. The second principal component (PC2), explaining 20.80% of the variance, is negatively correlated with the concentrations of oxalic, D-(−)-tartaric, fumaric, citric, maleic and propionic acid (Table S2). Four groups are clearly distinguished: three homogenous and one heterogenous. The first one is composed of all deciduous honeydew honey samples. The second group consists of all coniferous honeydew honey samples. Both groups include samples with high contents of D-(−)-quinic, D-(+)-malic, L-(+)-lactic, fumaric and succinic acids as well as the total content of acids. Although the organic-acid profiles of honeydew honeys appear largely comparable, the coniferous honeydew samples are characterized by higher concentrations of L-(+)-lactic acid. The third group includes 13 (out of 14) buckwheat honey samples that contained the highest amounts of D-(−)-tartaric (the only variety in which it was detected) and propionic acid. The rest of the samples are included in the fourth group (indicating low to moderate concentrations of each acid as well as low total content of acids) or not grouped in any of the four clusters (five samples).

PCA simplifies complex, multivariate datasets into interpretable principal components, enabling visual clustering and differentiation of honey varieties. In this study, we used 13 variables describing organic-acid contents. Our set of variables enabled rapid separation of three varieties, two types of honeydew and buckwheat honey, from the other honey varieties. Organic-acid composition was shown to be a useful trait for varietal honey authenticity confirmation by several authors (e.g., [11,12,13]). In these aforementioned studies, the contents of 5 to 22 organic acids were included in the PCAs. However, combining diverse chemical and physicochemical features often yields clearer varietal separation than using single parameters alone (e.g., [58,59]). In our previous study, we demonstrated that phenolic compound profiles, together with antioxidant properties, enabled clear discrimination between dark and light honeys and further separation of buckwheat and heather honeys [31]. These findings underscored the significant role of phenolics in honey variety classification. Although phenolic compounds were not assessed in the present work, our results indicate that organic-acid profiles also provide possible markers for differentiating honey varieties. It can be assumed that the integration of these parameters—organic acids, phenolics and antioxidant properties—together with additional physicochemical features such as pH, free acidity, sugar composition, etc., would further enhance the resolution of varietal differentiation, particularly among nectar honeys.

## 3. Materials and Methods

### 3.1. Chemicals and Reagents

An organic-acid standard kit and other reagents were of analytical standard grade or gradient grade. Organic-acid kits (oxalic, D-(−)-tartaric, D-(−)-quinic, formic, D-(+)-malic, malonic, L-(+)-lactic, citric, fumaric, succinic, maleic and propionic acids) were obtained from Supelco (Ballefonte, PA, USA). A stock standard containing the 12 organic acids indicated above was prepared. For buffering the mobile phase and adjusting the pH to 2.9, potassium phosphate monobasic (J.T.Baker Ultrapure Bioreagent for Liquid Chromatography from J.T.Baker, S.Witko, Łódź, Poland) and 85–90% phosphoric acid of HPLC grade (Fluka Analytical, Sigma-Aldrich from Merck, Darmstadt, Germany) were used. Conductivity standards of 147 µS/cm; 1413 µS/cm (Hamilton, Switzerland) and 0.05028 S/m; and 0.141116 S/m (LabStand, Poland) were obtained from Alchem, Poland. Cellulose membrane filters Chromafil CA-45/25, 0.45 µm were purchased from Macherey-Nagel GmbH 7 Co. KG (Düren, Germany).

Ultrapure water, for both solvent and diluent, from the Milli-Q system (Barnstead, Dubuque, IA, USA; resistivity: 18.3 MΩ cm) was used.

### 3.2. Honey Samples

Honey samples were obtained from apiaries maintained by the Apicultural Division of the National Institute of Horticultural Research in Poland and from privately owned apiaries from different regions of Poland (Lublin Upland, Upper Silesia, Lower Silesia, Greater Poland, Lesser Poland, Pomerania, Mazovia and Masuria). From 2017 to 2021, a total of 194 honey samples were collected. The final results are shown for 152 samples (see the justification in subchapter 3.4).

Microscopic pollen analysis was used to determine the botanical origins of the nectar honey samples. The analysis was carried out in accordance with the recommendations of the International Commission of Bee Botany according to the previously described procedure, with slight modifications [60,61]. The principle of this method was to determine the percentage of pollen grains of individual plant species in the honey sediment under a microscope. At least 300 consecutive pollen grains of nectariferous plants were determined on microscopic slides using an Olympus BX41 microscope (Olympus America, Center Valley, PA, USA) at 400× magnification. Then, the relative frequency of each pollen type was calculated as its percentage of the total count of pollen grains from nectariferous species. Three repetitions were made for each honey sample, and the final result was presented as a mean value. Honey samples containing at least 45% pollen grains of a particular type were qualified as monofloral. For acacia honey, a minimum of 30% *Robinia pseudoacacia* pollen grains was considered sufficient for classification as monofloral, while for linden honey, a minimum of 20% *Tilia* pollen grains was required.

Electrical conductivity measurements were performed for honeydew honey classification using a WTW inoLAB Cond 700 conductometer (WTW, Weilheim, Germany) according to the method elaborated upon by Szczęsna & Rybak-Chmielewska [62]. That method is based on measuring the electrical conductivity of a 20% (*w*/*w*, dry-weight basis) honey solution at 20 °C. The results were recalculated for a temperature of 20 °C using a temperature correction factor of 2.6% per 1 °C. Three repetitions were made for each honey sample, and the final results were expressed as an average of the results. The tested honey samples were classified as deciduous honeydew honey if the results of the electrical conductivity were from 0.80 to 0.95 mS/cm and as honeydew honey from coniferous trees if the electrical conductivity was over 0.95 mS/cm [32].

The honey samples were stored for up to one month at −20 °C until analysis.

### 3.3. HPLC-DAD Analysis

#### 3.3.1. Instrumentation and Conditions

A 5.00 g honey sample was dissolved in ultrapure water, diluted to 50 mL, mixed to ensure complete dissolution, filtered through cellulose membrane filter (0.45 µm) and injected directly onto the chromatographic column.

Chromatographic analyses of organic acids were carried out using a high-performance liquid chromatography system (HPLC) (Prominence, SHIMADZU CO., Kyoto, Japan) consisting of a Shimadzu LC-20AD Pump, DGU-20A 3R degassing unit, CTO-20AC Column Oven, SIL-10A HT Autosampler and SPD-M20A Diode Array Detector. The organic acids were separated on a Synergi Hydro-RP 80Ä C18 column (250 × 4.6 mm, 4 µm) (Phenomenex Inc., Torrance, CA, USA) at 25 °C. Extremely polar analytes are often poorly retained and insufficiently separated on conventional C18 columns. The Synergi Hydro-RP column features a C18 bonded phase that is endcapped with a unique proprietary polar group, providing enhanced retention for both hydrophobic and highly polar compounds. This design enables effective separation of extremely polar analytes, including organic acids, even under 100% aqueous conditions. The HPLC-DAD method parameters for organic-acid separation in honey are demonstrated in Table 4. The spectra of the individual organic acids were recorded at wavelengths ranging from 190 to 400 nm. The optimum wavelength for the simultaneous determination of the organic acids chosen for further research was 220 nm. LabSolutions software ver. 5.127 SP1 (Shimadzu, Japan) was used for the control, collection, processing and analysis of the HPLC-DAD results.

#### 3.3.2. Method Validation

Validation of the HPLC-DAD method for organic-acid analysis was carried out by evaluation of the following method parameters: linearity range, limit of detection and quantification, precision (CV) and accuracy calculated as recovery of the HPLC assay.

The organic-acid peaks were identified based on the comparison of individual acids’ retention times of the reference and the analyzed honey solution. For quantification of organic acids, the external calibration curve was calculated by the analysis of the working standard solutions at the following concentration levels: 1.25, 2.50, 3.75 and 5.00 µg/mL for oxalic acid; 6.25, 12.50, 18.75 and 25.00 µg/mL for citric and D-(−)-tartaric acid; 12.50, 25.00, 37.50 and 50.00 µg/mL for D-(−)-quinic, formic, D-(+)-malic, malonic, L-(+)-lactic, succinic and propionic acid; and 0.025, 0.05, 0.075 and 0.10 µg/mL for fumaric and maleic acid (Figure S3). Peak analysis was performed using LabSolutions software, which enables the analysis of photodiode array detector (DAD) data to separate and quantify overlapping peaks in HPLC chromatograms. In this study, peak deconvolution was applied to identify and quantify organic acids in varietal honey samples. The results demonstrated that the organic acids detected in the analyzed honeys did not exhibit overlapping peaks with other compounds. An example chromatogram of organic acids after deconvolution is shown in Figure S2.

The linearity range was estimated by plotting the peak area corresponding to each of the analyzed organic acids against the analyte concentration and then using the least-squares method for calculation of the respective regression coefficient and the coefficient of determination (R^2^). The precision of the method was expressed as the coefficient of variation (CV), and it was evaluated by successive determination of the sample solutions, using six independent repetitions, on the same working day. The recovery study was determined by spiking the honey samples with known concentrations of analytes (+50% of an analyte amount determined in an initial measurement of the same but non-spiked sample). The limit of detection (LOD) and limit of quantification (LOQ) for each of the analyzed acids were calculated by taking into account the residual standard deviation (SD) of the analytical signal and slope of the calibration curve (s), according to the formulas LOD = 3.3 SD/s and LOQ = 10 SD/s, respectively.

### 3.4. Statistical Analyses

For the nondetects, a value of LOD/2^0.5^ was substituted to avoid imputation of zero values [63]. From the initial set of 194 samples, those with acid contents below Q_1_ − 1.5 IQR or above Q_3_ + 1.5 IQR were discarded from each honey variety data subset. This approach was applied when outliers were found in at least 30% of the measurements for a particular honey sample. The final number of samples subjected to statistical analysis was 152. Data normality was assessed using the Shapiro–Wilk test. As the assumption of normal distribution was not met, the differences in organic-acid content among the honey varieties were evaluated using the Kruskal–Wallis test. Cluster analysis was conducted to identify hierarchical relationships, with results visualized as dendrograms combined with heatmaps using the Heatmapper tool [64]. Principal component analysis (PCA) was applied to illustrate variability in the organic-acid composition among honey varieties. All statistical analyses were performed using Statistica, version 13.3 (TIBCO Software Inc., Palo Alto, CA, USA).

## 4. Conclusions

There were significant botanical origin-related differences in the organic-acid content among the tested samples of honey. Oxalic, D-(−)-quinic, formic, citric and propionic acid were present in all honey varieties. L-(+)-lactic acid was found in all honey varieties except linden honey, and the highest content of the acid was quantified in coniferous honeydew (>2000 mg/kg), followed by deciduous honeydew honeys (ca. 850 mg/kg). Succinic acid was detected only in honeydew honeys and D-(−)-tartaric acid- only in buckwheat honey. The deciduous and coniferous honeydew honeys were characterized by the highest contents of organic acids (ca. 1500 and over 2500 mg/kg, respectively), followed by buckwheat honey (ca. 500 mg/kg). In contrast, the total organic-acid content of the acacia and rape honeys was the lowest (<200 mg/kg). The hierarchical clustering method clearly separated the coniferous and deciduous honeydew honeys from the other samples. Further, the PCA showed clear distinction of honeydew honeys (separate groups for coniferous and deciduous honeydew) and buckwheat honeys from the other varieties. Our study suggests that high content of L-(+)-lactic acid and the presence of succinic acid in honeydew honeys as well as the presence of D-(−)-tartaric acid in buckwheat honeys may be considered in further studies on chemical markers of the authenticity of these varieties.

Our findings on the organic-acid composition of varietal honeys extend the current understanding of the physicochemical characteristics of honeys produced under the climatic conditions of Poland. While the results obtained from our samples are promising, further research and validation on a larger set of samples—ideally encompassing diverse geographical regions—are required to reliably identify potential markers for the differentiation of honey varieties.

## Figures and Tables

**Table 2:** HPLC-DAD method validation parameters for the organic acids in honey.

Name of Acid	Linear Range (µg/mL)	R^2^	LOD (µg/mL)	LOQ (µg/mL)	CV (%)	Recovery (%)
Oxalic	1–5	0.9996	0.10	0.31	8.27	96.47
D-(−)-tartaric	6.25–25	0.9994	0.40	1.20	6.35	98.26
D-(−)-quinic	12.5–50	0.9998	1.06	3.20	5.02	98.45
Formic	12.5–50	0.9994	3.74	11.33	4.98	95.28
D-(+)-malic	12.5–50	1.0000	3.98	12.06	6.47	91.42
Malonic	12.5–50	0.9996	0.94	2.83	9.12	96.24
L-(+)-lactic	12.5–50	0.9997	2.00	6.07	6.28	97.56
Citric	6.25–25	0.9997	0.99	3.01	6.38	98.13
Fumaric	0.025–0.1	0.9997	0.03	0.08	4.69	92.37
Succinic	12.5–50	0.9994	2.60	7.89	4.57	98.68
Maleic	0.025–0.1	0.9995	0.01	0.03	3.25	96.56
Propionic	12.5–50	0.9998	4.45	13.50	8.24	95.74

R^2^—coefficient of determination, LOD—limit of detection, LOQ—limit of quantification, CV—coefficient of variation.

**Table 3:** Content (mg/kg) of organic acids in Polish varietal honeys. Mean ± standard deviation is given (in bold) along with coefficient of variation expressed as a percentage (in brackets).

Honey Variety (No. of Samples)	Oxalic Acid	D-(−)- Quinic Acid	Formic Acid	Citric Acid	Propionic Acid	D-(+)- Malic Acid	L-(+)- Lactic Acid	Fumaric Acid	Maleic Acid	Malonic Acid	Succinic Acid	D-(−)- Tartaric Acid	Sum of Acids
Goldenrod (*n* = 5)	**18.52 ± 3.56 ^A^** (19.20%)	**41.13 ± 15.97 ^A^** (38.82%)	**72.33 ± 13.06 ^B^** (18.05%)	**14.66 ± 6.08 ^B^** (41.49%)	**10.04 ± 6.19 ^A^** (61.59%)	**21.43 ± 10.71 ^B^** (49.99%)	**47.02 ± 25.28 ^AB^** (53.77%)	**0.09 ± 0.05 ^A^** (51.77%)	**0.01 ± 0.01 ^A^** (54.74%)	all samples < LOD	all samples < LOD	all samples < LOD	**227.74 ± 54.56 ^A^** (23.69%)
Willow (*n* = 9)	**21.92 ± 3.33 ^AB^** (15.18%)	**55.48 ± 8.35 ^AB^** (15.05%)	**41.84 ± 10.97 ^AB^** (26.21%)	**14.75 ± 7.85 ^B^** (53.22%)	**5.79 ± 3.40 ^A^** (58.78%)	**29.19 ± 9.26 ^B^** (31.72%)	**43.32 ± 20.94 ^AB^** (48.33%)	**0.25 ± 0.06 ^B^** (22.08%)	**0.02 ± 0.01 ^A^** (70.51%)	all samples < LOD	all samples < LOD	all samples < LOD	**212.75 ± 42.37 ^A^** (19.91%)
Acacia (*n* = 9)	**17.13 ± 0.36 ^A^** (2.13%)	**52.97 ± 3.11 ^A^** (5.87%)	**30.48 ± 2.80 ^A^** (9.19%)	**3.77 ± 0.68 ^A^** (18.09%)	**48.24 ± 25.84 ^BC^** (53.56%)	**9.18 ± 2.09 ^A^** (22.75%)	**36.91 ± 7.77 ^A^** (21.06%)	all samples < LOD	all samples < LOD	all samples < LOD	all samples < LOD	all samples < LOD	**198.94 ± 22.86 ^A^** (11.49%)
Buckwheat (*n* = 14)	**28.76 ± 6.76 ^B^** (23.50%)	**42.47 ± 11.79 ^A^** (27.76%)	**98.03 ± 23.11 ^BC^** (23.57%)	**37.61 ± 7.38 ^BC^** (19.62%)	**82.49 ± 22.54 ^C^** (27.32%)	**86.21 ± 18.23 ^BC^** (21.15%)	**100.62 ± 41.42 ^B^** (41.17%)	**0.42 ± 0.15 ^BC^** (36.44%)	**0.08 ± 0.02 ^B^** (27.72%)	all samples < LOD	all samples < LOD	**22.17 ± 5.05** (22.80%)	**499.00 ± 103.67 ^B^** (20.78%)
Linden (*n* = 23)	**19.6 ± 1.76 ^AB^** (8.99%)	**50.62 ± 10.29 ^A^** (20.32%)	**40.09 ± 14.17 ^AB^** (35.34%)	**12.52 ± 4.34 ^B^** (34.68%)	**3.37 ± 2.53 ^A^** (75.06%)	**23.53 ± 6.43 ^B^** (27.32%)	all samples < LOD	**0.21 ± 0.04 ^AB^** (16.74%)	**0.01 ± 0.01 ^A^** (55.03%)	**63.65 ± 24.39 ^B^** (38.33%)	all samples < LOD	all samples < LOD	**213.76 ± 32.58 ^A^** (15.24%)
Phacelia (*n* = 32)	**17.4 ± 2.23 ^A^** (12.81%)	**82.61 ± 13.55 ^B^** (16.40%)	**54.65 ± 7.59 ^AB^** (13.88%)	**8.86 ± 3.39 ^AB^** (38.23%)	**3.80 ± 2.54 ^A^** (66.96%)	**17.01 ± 7.82 ^AB^** (45.98%)	**47.71 ± 27.78 ^AB^** (58.23%)	**0.12 ± 0.07 ^A^** (56.06%)	**0.02 ± 0.01 ^A^** (46.85%)	all samples < LOD	all samples < LOD	all samples < LOD	**232.58 ± 39.6 ^A^** (17.02%)
Rape (*n* = 29)	**15.31 ± 1.31 ^A^** (8.53%)	**46.81 ± 14.15 ^A^** (30.22%)	**39.80 ± 8.27 ^A^** (20.77%)	**25.55 ± 5.96 ^BC^** (23.35%)	**3.01 ± 1.85 ^A^** (61.27%)	**5.29 ± 3.82 ^A^** (72.13%)	**18.93 ± 12.37 ^A^** (65.35%)	**0.09 ± 0.03 ^A^** (37.40%)	**0.01 ± 0.01 ^A^** (40.92%)	**1.85 ± 1.10 ^A^** (59.44%)	all samples < LOD	all samples < LOD	**156.84 ± 24.73 ^A^** (15.77%)
Coniferous honeydew (*n* = 15)	**22.89 ±** **4.23 ^B^** (6.43%)	**140.39 ± 33.35 ^C^** (23.56%)	**84.98 ± 15.64 ^BC^** (14.25%)	**22.52 ± 18.91 ^BC^** (31.88%)	**16.47 ± 8.58 ^AB^** (11.47%)	**135.32 ± 73.40 ^C^** (15.08%)	**2162.49 ± 678.10 ^D^** (9.35%)	**1.45 ± 0.53 ^C^** (15.71%)	**0.04 ± 0.03 ^AB^** (28.35%)	all samples < LOD	**10.82 ± 6.78 ^A^** (43.80%)	all samples < LOD	**2597.48 ± 603.39 ^C^** (8.33%)
Deciduous honeydew (*n* = 11)	**29.31 ± 3.93 ^B^** (13.40%)	**182.73 ± 10.11 ^C^** (5.53%)	**100.76 ± 15.30 ^C^** (15.19%)	**44.9 ± 22.05 ^C^** (49.11%)	**25.52 ± 11.04 ^B^** (43.24%)	**233.28 ± 81.43 ^C^** (34.91%)	**854.18 ± 208.78 ^C^** (24.44%)	**1.73 ± 0.74 ^C^** (42.94%)	**0.10 ± 0.02 ^B^** (22.54%)	all samples < LOD	**19.47 ± 5.91 ^A^** (30.35%)	all samples < LOD	**1492.09 ± 302.86 ^BC^** (20.30%)
Heather (*n* = 5)	**22.19 ± 5.37 ^AB^** (24.21%)	**47.16 ± 17.45 ^A^** (37.01%)	**75.05 ± 22.26 ^BC^** (29.66%)	**16.22 ± 10.94 ^B^** (67.42%)	**7.78 ± 4.51 ^A^** (57.91%)	all samples < LOD	**38.24 ± 24.71 ^A^** (64.62%)	**0.11 ± 0.07 ^A^** (63.92%)	**0.04 ± 0.02 ^AB^** (55.82%)	**60.38 ± 36.24 ^B^** (60.02%)	all samples < LOD	all samples < LOD	**270.78 ± 107.09 ^A^** (39.55%)
H	103.866	112.351	113.049	113.588	95.757	129.336	126.972	113.336	108.888	42.138	10.540	-	116.789
p	<0.001	<0.001	<0.001	<0.001	<0.001	<0.001	<0.001	<0.001	<0.001	<0.001	0.001	-	<0.001

SD—standard deviation, CV—coefficient of variation, LOD—limit of detection. Values that are significantly different according to the Kruskal–Wallis test are marked with different letters. They should be compared along the columns.

**Table 4:** Method parameters for organic acid separation by the HPLC-DAD technique.

Feature	Characteristics
Elution mode	Isocratic
Column	Synergi Hydro-RP 80Ä C18 column (250 × 4,6 mm, 4 µm) (Phenomenex Inc., Torrance, CA, USA)
Mobile phase	20 mM potassium phosphate (pH 2.9)
Analysis time	15 min; wash/equilibration time = 15 min
Flow rate	0.7 mL/min
Oven temperature	25 °C
DAD range (UV detection)	190 to 400 nm (220 nm)
Injection volume	20 µL

## Data Availability

The original contributions presented in this study are included in the article/Supplementary Materials. Further inquiries can be directed to the corresponding author.

## Supplementary Materials

The following supporting information can be downloaded at: https://www.mdpi.com/article/10.3390/molecules30214261/s1, Figure S1: HPLC-DAD chromatogram of standards; Table S1: Eigenvalues and the proportion of variation (%) explained by the principal components; Table S2: Correlations between the principal components and the original variables; Figure S2: Chromatogram of organic acids in a rapeseed honey sample after deconvolution; Figure S3: Calibration curves of the studied organic acids.
