# Supplementary material for: Qualitative and Quantitative Characteristics of Organic Acids in Monofloral and Honeydew Honeys from Poland: Is There a Varietal Pattern in Their Composition?
Source: Molecules. 2025 Oct 31;30(21):4261. doi: 10.3390/molecules30214261 (PMC12608658; doi:10.3390/molecules30214261)
Supplement: Supplementary file 1 [file molecules-30-04261-s001.zip › molecules-3914464-supplementary.pdf]

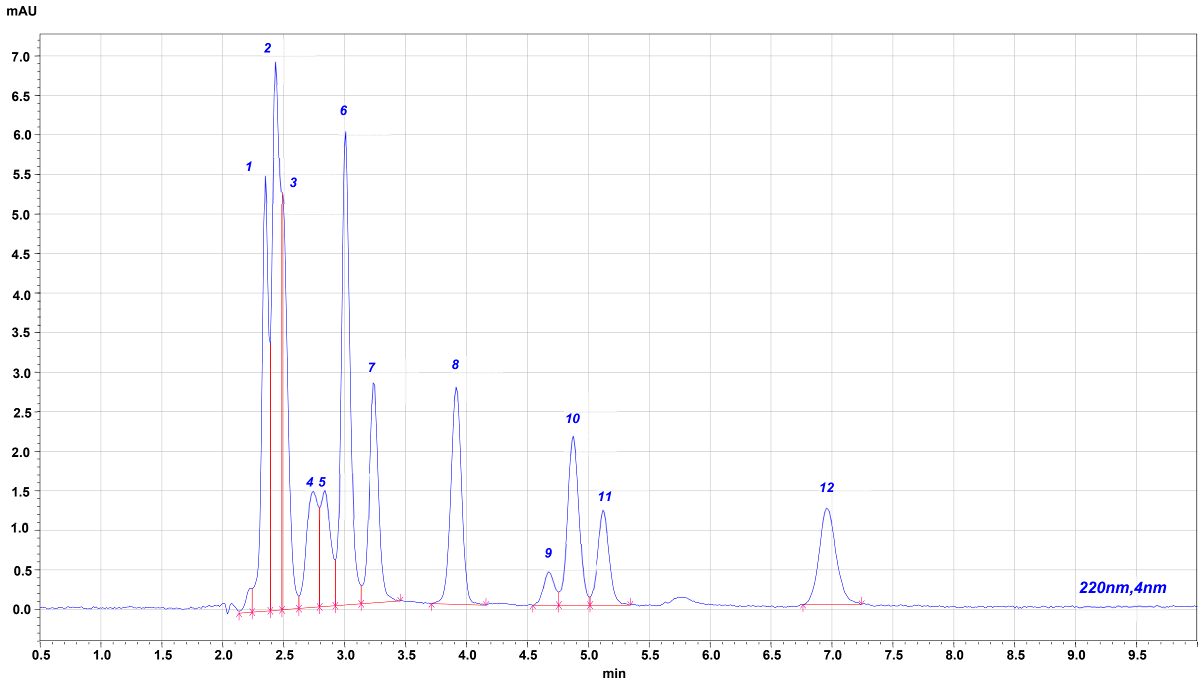

Figure S1. HPLC-DAD chromatogram of standards. The peaks correspond to the following: (1) oxalic acid;(2) D-(-)-tartaric acid; (3) D-(-)-quinic acid; (4) formic acid; (5) D-(+)-malic acid; (6) malonic acid; (7) L-(+)-lactic acid; (8) citric acid; (9) fumaric acid; (10) succinic acid; (11) maleic acid; (12) propionic acid.

Table S1. Eigenvalues and the proportion of variation (%) explained by the principal components.

Component	Eigenvalue	Proportion (%)	Cumulative (%)
1	6.96	53.58	53.58
2	2.70	20.80	74.38
3	1.04	8.00	82.37
4	0.74	5.71	88.08
5	0.52	4.01	92.10
6	0.34	2.58	94.68
7	0.27	2.05	96.73
8	0.14	1.09	97.82
9	0.13	0.98	98.80
10	0.08	0.60	99.39
11	0.06	0.44	99.83
12	0.02	0.17	100.00

Table S2. Correlations between the principal components and the original variables.

Variable	Principal component		
	1	2	3
Oxalic acid	-0.766	-0.407	-0.276
D-(-)-Tartaric acid	-0.291	-0.903	0.065
D-(-)-Quinic acid	-0.777	0.470	0.048
Fumaric acid	-0.836	-0.298	-0.065
D-(+)-Malic acid	-0.943	0.083	-0.055
Malonic acid	0.245	0.032	-0.948
L-(+)-Lactic acid	-0.760	0.446	0.097
Citric acid	-0.546	-0.381	0.027
Fumaric acid	-0.920	0.282	-0.057
Succinic acid	-0.863	0.372	-0.044
Maleic acid	-0.820	-0.388	-0.095
Propionic acid	-0.429	-0.745	0.157
Sum of acids	-0.847	0.370	0.049

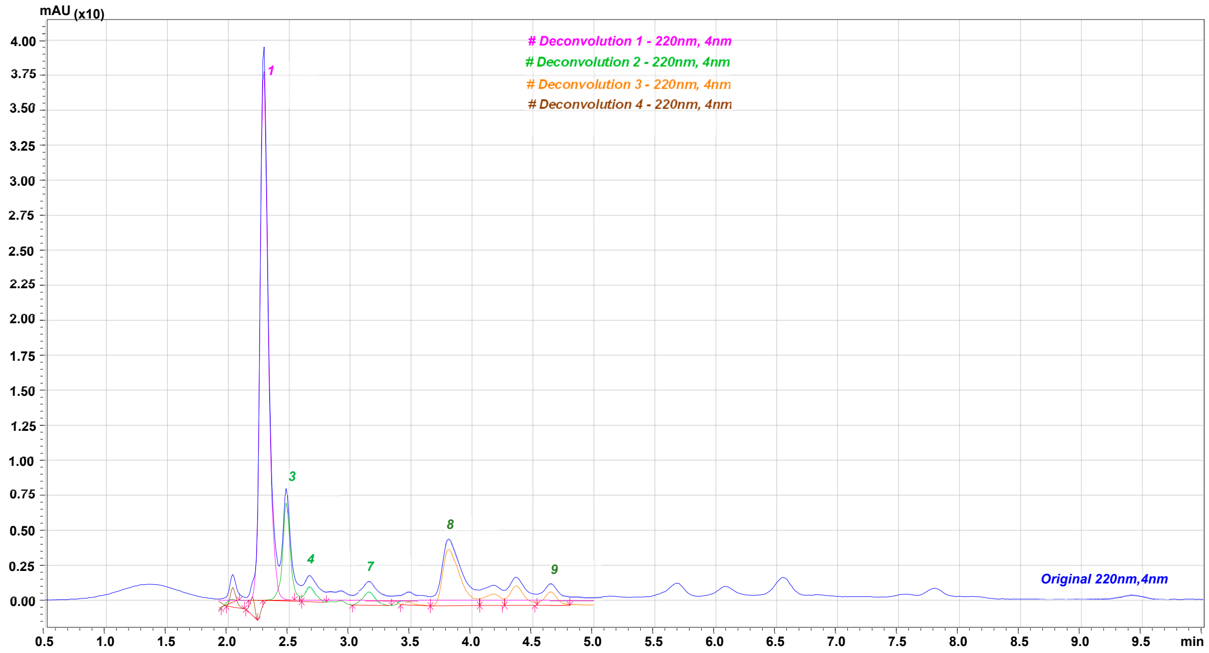

Figure S2. Chromatogram of organic acids in a rapeseed honey sample after deconvolution. The peaks correspond to the following: (1) oxalic acid; (3) D-(-)-quinic acid; (4) formic acid; (7) L-(+)-lactic acid; (8) citric acid; (9) fumaric acid.

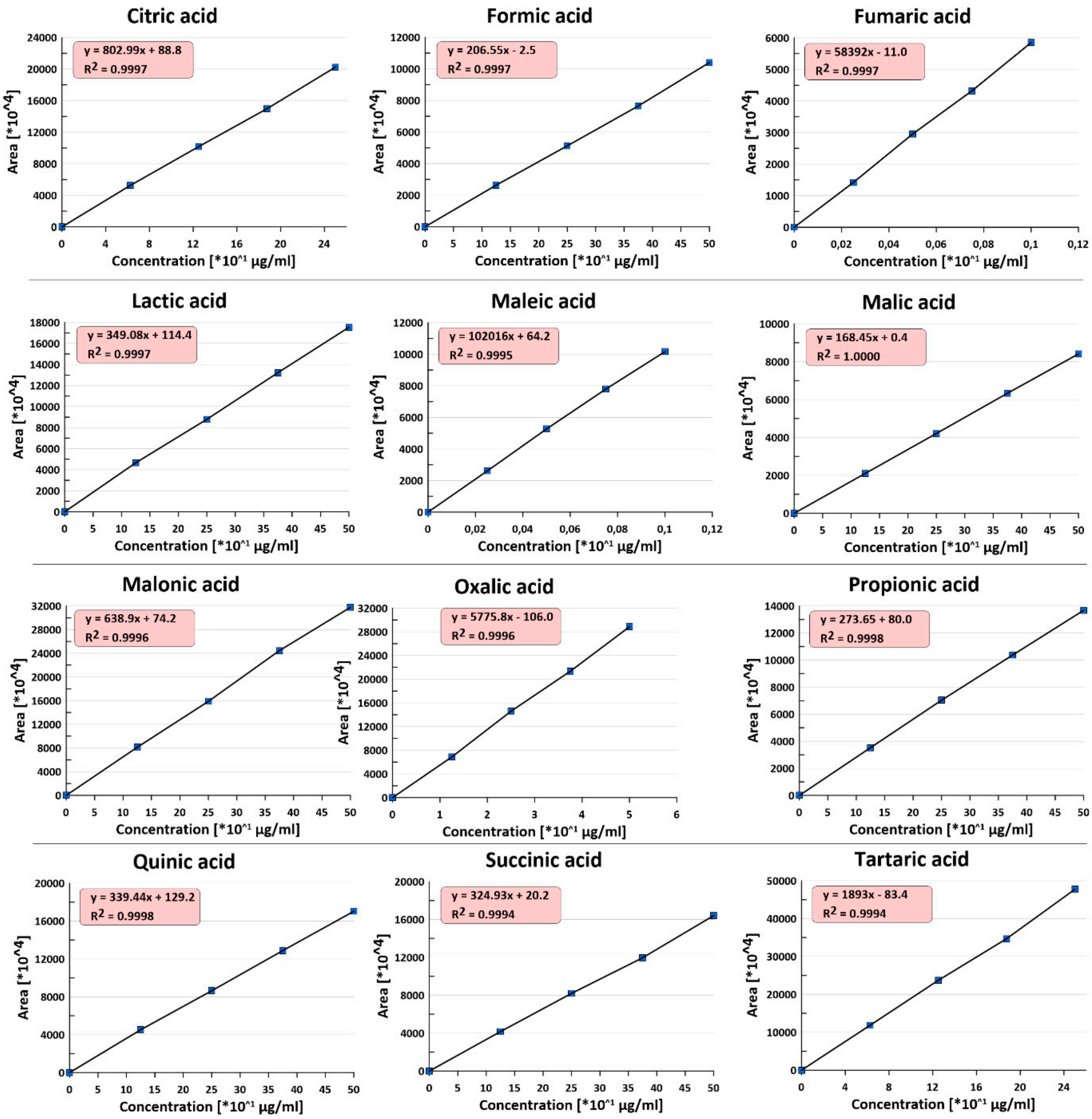

Figure S3. Calibration curves of the studied organic acids.
